# Evidence for nonallopatric speciation among closely related sympatric *Heliotropium* species in the Atacama Desert

**DOI:** 10.1002/ece3.929

**Published:** 2013-12-29

**Authors:** Federico Luebert, Pit Jacobs, Hartmut H Hilger, Ludo A H Muller

**Affiliations:** 1 Freie Universität Berlin, Institut für Biologie – BotanikAltensteinstraße 6, D-14195, Berlin, Germany; 2 Departamento de Silvicultura, Facultad de Ciencias Forestales y Conservación de la Naturaleza, Universidad de ChileSantiago, Chile; Nees-Institut für Biodiversität der Pflanzen, Rheinische Friedrich-Wilhelms-UniversitätMeckenheimer Allee 170, D-53115, Bonn, Germany

**Keywords:** Adaptive radiation, allopatry, Chile, *Heliotropium*, speciation, sympatry

## Abstract

The genetic structure of populations of closely related, sympatric species may hold the signature of the geographical mode of the speciation process. In fully allopatric speciation, it is expected that genetic differentiation between species is homogeneously distributed across the genome. In nonallopatric speciation, the genomes may remain undifferentiated to a large extent. In this article, we analyzed the genetic structure of five sympatric species from the plant genus *Heliotropium* in the Atacama Desert. We used amplified fragment length polymorphisms (AFLPs) to characterize the genetic structure of these species and evaluate their genetic differentiation as well as the number of loci subject to positive selection using divergence outlier analysis (DOA). The five species form distinguishable groups in the genetic space, with zones of overlap, indicating that they are possibly not completely isolated. Among-species differentiation accounts for 35% of the total genetic differentiation (*F*_ST_ = 0.35), and *F*_ST_ between species pairs is positively correlated with phylogenetic distance. DOA suggests that few loci are subject to positive selection, which is in line with a scenario of nonallopatric speciation. These results support the idea that sympatric species of *Heliotropium* sect. *Cochranea* are under an ongoing speciation process, characterized by a fluctuation of population ranges in response to pulses of arid and humid periods during Quaternary times.

## Introduction

Based on the spatial segregation of the involved populations, three major geographical categories have traditionally been recognized for the process of speciation. In the case of allopatric speciation, geographically isolated populations differentiate into distinct species and the speciation event occurs in the absence of gene flow. Sympatric speciation, on the other hand, is a process that occurs in a single (geographical) population and in the presence of gene flow. Besides these two extreme forms of spatial segregation, partial segregation of the diverging populations can occur. The diversification of such populations with adjacent geographical ranges into distinct species, despite limited interbreeding at their contact zone, characterizes the process of parapatric speciation.

Classification of the speciation process into the discrete allopatric, parapatric, and sympatric modes is now considered an oversimplification by several authors who have argued that speciation studies should focus on modeling and estimating parameters that describe the diversification process rather than on classifying the speciation mode according to geographical criteria (e.g., Wu 2001; Butlin et al. 2008; Fitzpatrick et al. 2008; Coyne 2011). Nevertheless, theoretical models have illustrated the possibility of sympatric speciation (Bolnick and Fitzpatrick 2007), and its occurrence in nature has been shown in several studies (e.g., Barluenga et al. 2006; Savolainen et al. 2006; Papadopulos et al. 2011). The relative scarcity of reports on the occurrence of speciation with gene flow in nature is thereby likely due to the fact that allopatric speciation is commonly accepted as the null model (Coyne and Orr 2004) and nonallopatric alternatives are usually not considered in speciation studies (Johannesson 2010). Despite this apparent bias in the treatment of allopatric and sympatric speciation models, the results of Papadopulos et al. (2011) show that sympatric speciation may be more common than previously accepted and highlight the relevance of gene flow between incipient species (Wu 2001; Fitzpatrick et al. 2009; Coyne 2011). In order to comply with the current views on the different modes of speciation, in this study, we refer to sympatric speciation as a speciation process that started in the presence of panmixia.

Sympatry or co-occurrence of closely related species can either result from a sympatric speciation process or from secondary contact due to range expansion after speciation. Theoretical models as well as empirical studies have shown that the genetic structure of closely related sympatric species holds a signature of the geographical mode of the speciation process (e.g., Charlesworth et al. 1997; Wilding et al. 2001; Savolainen et al. 2006; Nosil et al. 2009; Strasburg et al. 2012; Via 2012). Under the allopatric scenario, genetic variation tends to be uniform across the genome due to a large proportion of the genome changing through a combination of divergent selection, differential response to similar selective pressures and genetic drift (Nosil et al. 2009; Strasburg et al. 2012). In contrast, in the extreme case of sympatric speciation, gene flow between the incipient species can homogenize most of the genome, except for the loci that experience strong divergent selection pressures or regions that are tightly linked with these loci (Charlesworth et al. 1997; Strasburg et al. 2012; Via 2012; Via et al. 2012). Closely related species currently living in local sympatry thus provide an opportunity to examine these models empirically and to infer possible modes of speciation. However, closely related species tend to have similar ecological characteristics, and their sympatry is constrained by competition (HilleRisLambers et al. 2012). For this reason, cases of clades containing multiple sympatric and closely related species are rare.

In the Atacama Desert, there are at least two documented groups of closely related plant species that are to a large extent sympatric: *Nolana* (Dillon et al. 2009) and *Heliotropium* (Luebert and Wen 2008; Luebert 2013). All but one of the *Heliotropium* species that inhabit the Atacama Desert belong to the monophyletic section *Cochranea* (Miers) I.M.Johnst. (Luebert and Wen 2008). This is one of the most speciose groups of the Atacama Desert, with all of its 17 species inhabiting that area (Luebert 2013). Phylogenetic studies are in line with the idea that *Heliotropium* sect. *Cochranea* underwent an adaptive radiation toward the early Pliocene in response to the aridification of the Atacama Desert (Luebert and Wen 2008; Luebert et al. 2011a,b2011b). However, sister relationships are largely unresolved, and little is known about the ecological and genetic forces as well as the spatial and temporal dynamics implied in its diversification. Incipient speciation is likely common in this group. Based on the ideas of Stebbins (1952) for speciation in arid zones, Luebert and Wen (2008) postulated that late Neogene and Quaternary diversification of *Heliotropium* sect. *Cochranea* may have occurred along with temporal pulses of spatial contraction and expansion of populations, generating alternating periods of isolation and reunion of diverging populations. This model of diversification implies allopatric speciation, because genetic isolation would occur during the contraction phases, and is consistent with long-term fluctuations of aridity in the Atacama Desert (Latorre et al. 2006). In fact, most evolutionary studies on Atacama Desert plant groups suggest a predominance of allopatric speciation (Gengler-Nowak 2002; Luebert et al. 2009; Viruel et al. 2012). Present distributions in *Heliotropium* sect. *Cochranea* show two major centers of diversity with, respectively, eight and six of 17 species sharing the same geographical area, and up to 6 species can occur in local sympatry within these areas. If the model postulated by Luebert and Wen (2008) holds, sympatry would have been achieved through secondary contact. Under this scenario, we would expect absence of gene flow among sympatric species associated with uniform genetic divergence across the genome. Under a scenario of sympatric speciation, however, we expect to find genetically differentiated populations with zones of genetic contact between species reflecting various levels of gene flow. Genetic divergence between species is then expected to occur in only a few loci that are subject to selection. However, depending on the time frame of allopatry, the pattern of gene flow may be very difficult to distinguish from the pattern expected under a scenario of speciation with gene flow (Strasburg et al. 2012).

In this study, we evaluate the genetic differentiation among five sympatric and closely related species of the plant genus *Heliotropium* L. (Heliotropiaceae) in the Atacama Desert using amplified fragment length polymorphism (AFLP) data. The following questions are addressed:

1. Are closely related and sympatric species of *Heliotropium* genetically isolated?
2. Is phylogenetic relatedness of *Heliotropium* species associated with gene flow?
3. Can a signature of allopatric, sympatric, or ongoing divergence with secondary gene flow in sympatry be found in the genetic structure of the *Heliotropium* species?

## Material and Methods

### Plant material and DNA extraction

The main study site is located in the area around the village of Totoral (S27°54′5″, W70°57′34″) in the coastal Atacama Desert of northern Chile. For population genetic analyses, we aimed at collecting leaves of at least 15 specimens and one voucher herbarium specimen of each of the following *Heliotropium* species: *Heliotropium filifolium* (Miers) I.M.Johnst., *H. floridum* (A.DC.) Clos, *H. longistylum* Phil., *H. megalanthum* I.M.Johnst., and *H. sinuatum* (Miers) I.M.Johnst. These are morphologically defined entities according to the species concepts of Luebert (2013). A total of five individuals of these species from the neighbor locality of Carrizal Bajo (S28°4′54″, W71°8′49″) was also included in the study. Samples were collected in the austral spring in 2003, 2005, and 2011 (see Table S1), and leaf material was dried and stored in silica gel until further processing. DNA was isolated using the Nucleospin Plant® II Kit (Macherey-Nagel GmbH, Düren, Germany) following manufacturer instructions.

### Amplified fragment length polymorphism

Amplified fragment length polymorphism was used to evaluate genetic differentiation within and between the species under study. AFLP analysis followed the protocol of Vos et al. (1995). Restriction enzymes *Eco*RI and *Mse*I were employed for digesting extracted DNA, and preamplification was performed using oligonucleotide primers *Eco*RI + A / *Mse*I + C. Three different primer combinations were used for the selective amplification: *Eco*RI + ATG / *Mse*I + CAG, *Eco*RI + ATA / *Mse*I + CAC, and *Eco*RI + ATT / *Mse*I + CAA. The selective *Eco*RI primers were labeled at their 5′-end with 6-carboxyfluorescein (6-FAM), and the selective PCR products were separated by electrophoresis using the Applied Biosystems DNA Analyzer 3730 (fragment analysis was performed by GATC Biotech AG (Konstanz, Germany)). Fragment length estimation and allele scoring were performed in GeneMarker® v1.95 (Softgenetics, State College, PA) using the GS500 size standard and the default settings of the AFLP analysis parameters, except for the minimum intensity of the peak detection threshold, which was increased from 100 to 500. AFLP fragments of the same size generated in different specimens were assumed to be homologous, and the relative intensities of the fragments were not considered. Loci for which the minor allele frequency (MAF) was at least 3% were considered polymorphic, and only the polymorphic loci were retained for further analysis. Reproducibility of the AFLP method was assessed by comparing replicate analyses of three specimens of each of the species *H. longistylum*,*H. filifolium,* and *H. megalanthum*.

### Data analysis

The level of linkage disequilibrium (LD) between AFLP markers and its significance was assessed by calculating *r*^2^ with the package LDcorSV v1.2 in R v2.15.2 (R Development Core Team 2013). An exact test of LD was performed in Genepop v4.2 (No. of steps in Markov chain = 10,000, No. of dememorization steps* *=* *1000; Rousset 2008), using the subset of polymorphic AFLP markers in each species separately and a Bonferroni correction to obtain a global significance of 0.05. Genetic diversity within species was estimated as the percentage of polymorphic AFLP markers and was corrected for unequal sample sizes using the method of multiple random subsampling with a sample size of seven and 50 subsamples (Leberg 2002). Genetic distances among individuals were calculated as pairwise asymmetric binary distances or Jaccard dissimilarities between individual samples (see e.g., Kosman and Leonard 2005) and visualized by multidimensional scaling (MDS) and hierarchical clustering (complete linkage) in R v2.15.2 with packages adegenet v1.3-8 (Jombart 2008) and ade4 v1.5-2 (Dray et al. 2007).

In order to evaluate the association between phylogenetic relatedness and gene flow, pairwise phylogenetic distances between species were calculated with the R package APE v3.0-8 (Paradis et al. 2004). A phylogenetic tree was generated by reanalyzing the sequence data obtained by Luebert and Wen (2008). These data consist of DNA sequences of three plastid regions (*ndhF, rps16, trnL-trnF*) and the nuclear ribosomal ITS region. In this dataset, each species of *Heliotropium* sect. *Cochranea*, except for *H*. *megalanthum, H*. *myosotifolium* (A.DC) Reiche, and *H*. *krauseanum* Fedde, is represented by two or more samples from different localities. For one additional individual of each of these three species, additional data (GenBank accessions Nrs. KF301622–KF301628) were generated by sequencing the same four markers following the protocols described by Luebert and Wen (2008). Information of sequences used in this study for phylogenetic analysis is detailed in Table S2. Sequence data were analyzed using a species-tree approach implemented in the software *BEAST v.1.6.1 (Heled and Drummond 2010). Each locus was analyzed using an uncorrelated lognormal relaxed clock model. Substitution models were specified according to results obtained from analyses with Modeltest v.3.7 (Posada and Crandall 1998). Modeltest uses the Akaike information criterion to select one among 24 possible substitution models with different numbers of parameters. Likelihoods of the alignment given a tree are calculated under each possible model using neighbor-joining trees. The final species tree was deposited in TreeBase (accession 14422).

Genetic differentiation within and among species was evaluated by analysis of molecular variance (AMOVA; Excoffier et al. 1992) using the R package pegas v0.4-4 (Paradis 2010) with pairwise Euclidean distances between the individual AFLP phenotypes and a permutation procedure (1000 permutations) to assess the significance of the different variance components. In our model, AMOVA partitioned the total genotypic variance into components due to differences between species and differences between specimens within species. Gene flow between species was estimated using the infinite island model as the effective number of migrants per generation *N*_e_*m* = (1−*F*_ST_)/(4*F*_ST_) (Wright 1949).

In order to detect loci under selection, divergence outlier analysis (DOA) was conducted with the software Mcheza (Antao and Beaumont 2011). Pairwise comparison of *F*_ST_ values between the species involved in this study was conducted with default settings. The subsample size and the expected number of total populations were, respectively, set to 0 and 2 for each species combination. The expected *F*_ST_ distribution under the null hypothesis of neutrality was calculated with 50,000 simulations and a false discovery rate of 0.1 within the 95% confidence interval in 10 different simulations. As calculation of the initial mean *F*_ST_ found in the dataset includes all potentially selected loci (i.e., loci falling outside of the confidence interval), those loci were removed from calculation of the “neutral” mean *F*_ST_. The mean simulated *F*_ST_ was forced to approximate the initial mean *F*_ST_ by application of a bisection algorithm over repeated simulations.

## Results

Amplified fragment length polymorphism analysis of 96 specimens belonging to five different *Heliotropium* species with three different selective PCR amplifications generated a total of 311 scorable loci, 287 (92%) of which were polymorphic across the whole set of samples. Per selective PCR, the number of polymorphic loci varied between 82 and 118 (with an average of 96; see Table 1). Reproducibility of the AFLP method was high, with an average allele call correspondence between replicates of 97%. Based on the set of 287 polymorphic loci, 96 different genotypes could be distinguished and the number of scored AFLP fragments per specimen varied between 72 and 154 (average of 120). Overall, linkage disequilibrium between AFLP markers was found to be low, with the average *r*^2^ value within species varying between 0.054 (*H. megalanthum*) and 0.17 (*H. floridum*; see Table 2) and the fraction of the marker pairs in significant linkage disequilibrium not exceeding 0.4% (data not shown). The percentage of polymorphic markers per species, corrected for unequal sample size, varied between 55% (*H. longistylum*,*H. filifolium,* and *H. megalanthum*) and 68% (*H. floridum*; see Table 2).

**Table 1:** Overview of oligonucleotide primer combinations used for the selective PCR amplification in the AFLP analysis of 96 specimens belonging to five different *Heliotropium* species and the numbers of polymorphic loci that were generated for each primer combination.

*Eco*RI +	*Mse*I +	# Polymorphic markers
ATG	CAG	87
ATA	CAC	82
ATT	CAA	118
Total		287

**Table 2:** Genetic diversities, estimated as percentages of polymorphic AFLP markers and corrected for unequal sample sizes by multiple random subsampling (subsample size = 7; Leberg 2002), in five *Heliotropium* species and average pairwise linkage disequilibria between polymorphic markers within each species measured as the square of the correlation coefficient between allele frequencies (*r*^2^); *N*, sample size; SD, standard deviation.

		% Polymorphic markers			
Species	*N*	Uncorrected	Corrected (SD)	*r*^2^ (SD)	*P*
*Heliotropium longistylum*	17	71	55 (3)	0.073 (0.12)	0.0000
*Heliotropium filifolium*	25	77	55 (5)	0.065 (0.14)	0.0042
*Heliotropium megalanthum*	21	72	55 (3)	0.054 (0.085)	0.0000
*Heliotropium sinuatum*	26	85	60 (7)	0.063 (0.11)	0.0000
*Heliotropium floridum*	7	68	NA	0.17 (0.21)	0.0000

**Figure 1:**
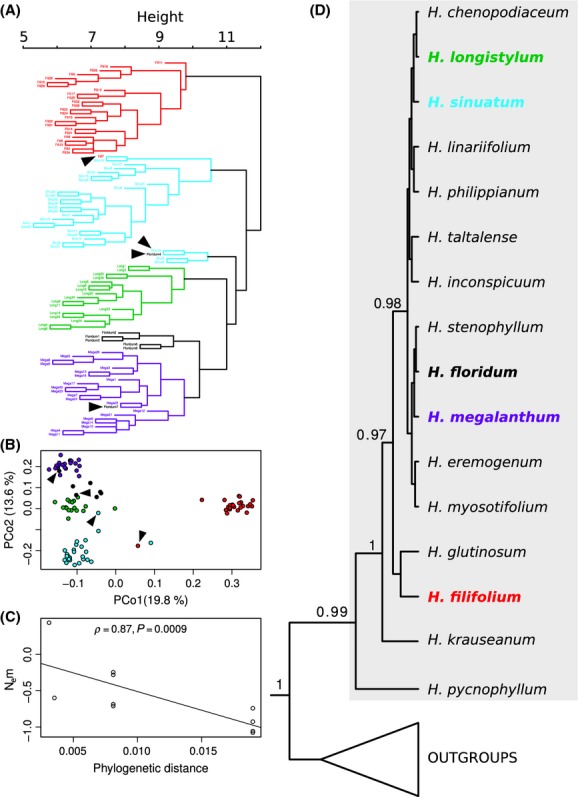
(A) Dendrogram resulted from cluster analysis. (B) Scatter plot of the two first axis (percentage of variance explained indicated in brackets) of the multidimensional scaling. (C) Relation between interspecific phylogenetic distance and N_e_m between populations, depicting the curve adjusted with a linear model and indicating the Spearman correlation and its *P*-value. (D) Phylogenetic analysis with Bayesian Posterior probabilities >50 above branches, *Heliotropium* sect. *Cochranea* highlighted in gray, and studied species in bold and color-coded. Color codes for species are the same across figures. Black arrows (A–B) indicate individuals of one species intermixed with individuals of other species.

Considering both the results of the hierarchical cluster analysis (Fig. 1A) and of the MDS (Fig. 1B), all species included in this study tended to form well-defined genetic groups. In some cases, however, specimens did not cluster according to their presumed taxonomic identity: one *H. filifolium* specimen clustered together with *H. sinuatum* and one *H. floridum* specimen clustered together with *H. megalanthum*.

Bayesian phylogenetic inference was conducted using the substitution models selected from Modeltest for each molecular marker: ITS (GTR+I+Γ), *trnL-trnF* (HKY+Γ), *rps*16 (GTR+Γ), ndhF (GTR+I+Γ). The resulting species tree (Fig. 1D) suggests that *Heliotropium pycnophyllum* and *H. krauseanum* are successive sister species to the remaining species of section *Cochranea*. Among the remaining species, *H. filifolium* and *H. glutinosum* appear in an unsupported sister relationship, while the other species form a well-supported clade, but with unresolved internal relationships. Most sympatric species in the study area fall in the latter, unresolved clade.

Estimated migration rates declined with increasing phylogenetic distance. Phylogenetic distances and *N*_e_*m* values among species showed a negative correlation (*ρ *= −0.87, *P *=* *0.0009; see Fig. 1C and Table S3). The same pattern was observed when comparing the number of private loci between species (data not shown).

Analysis of molecular variance indicated that approximately two-thirds (65%) of the total variability of the data can be explained by genetic variation among specimens within the investigated species, while 35% of the total variability is due to genetic differentiation between species (Table 3).

**Table 3:** Results of an AMOVA indicating sums of square deviations (SSD), mean square deviations (MSD), number of degrees of freedom, and variance components.

				Variance components		
	SSD	MSD	df	*σ*^2^	% variation	*P*-value
Populations	1539.909	384.97737	4	18.862	35.37	0
Error	3135.882	34.46024	91	34.460	64.63	
Total	4675.792	49.21886	95	53.322		

**Figure 2:**
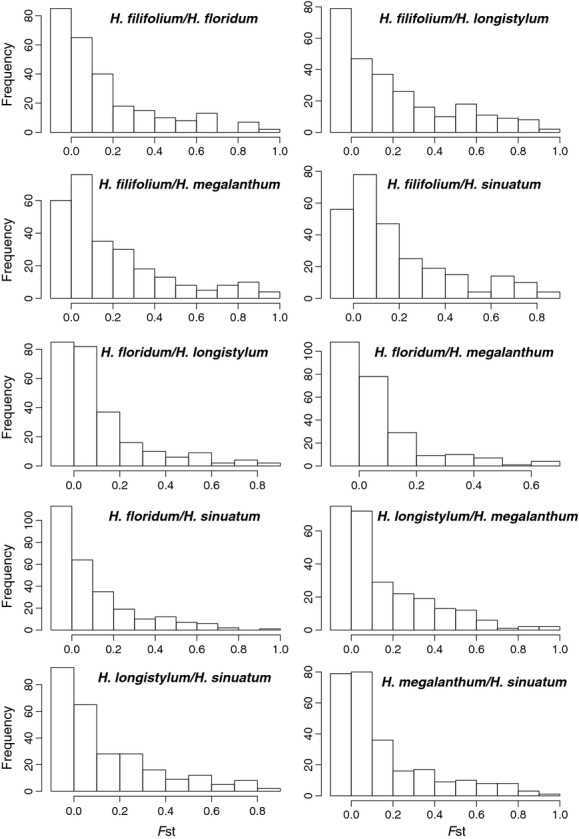
Histograms of *F*_*ST*_ frequency distribution for each species pair of sympatric species of *Heliotropium* sect. *Cochranea* in the area of Totoral, Chile.

Divergence outlier analysis with Mcheza showed that a variable but limited number of loci putatively under positive selection are detected in all pairwise comparisons of species (Table 4, Fig. S1). The distributions of frequencies of single-locus *F*_ST_ revealed a similar pattern. When *H. filifolium* was compared with *H. floridum*,*H. longistylum*, and *H. megalanthum,* a more even distribution could be observed (Fig. 2), indicating a higher level of differentiation (and therefore less gene flow). This is to be expected as *H. filifolium* is phylogenetically less related to the other species than the other species are to each other (Fig. 1D).

**Table 4:** Results of the divergence outlier analysis for each species pair. Percentage of outliers as calculated in relation to all polymorphic loci.

Species pair	Dataset *F*_ST_	“Neutral” *F*_ST_	*N* outliers	% outliers
*Heliotropium filifolium–Heliotropium floridum*	0.2489	0.205 ± 0.018	6 (0–9)	2.28
*Heliotropium filifolium–Heliotropium megalanthum*	0.3184	0.284 ± 0.016	8 (0–14)	3.04
*Heliotropium filifolium–Heliotropium longistylum*	0.3161	0.281 ± 0.034	4 (3–16)	1.52
*Heliotropium filifolium–Heliotropium sinuatum*	0.2791	0.256 ± 0.008	7 (7–8)	2.66
*Heliotropium floridum–Heliotropium megalanthum*	0.1004	0.086 ± 0.008	5 (5–7)	1.90
*Heliotropium floridum–Heliotropium longistylum*	0.1771	0.151 ± 0.009	4 (4–7)	1.52
*Heliotropium floridum–Heliotropium sinuatum*	0.1729	0.137 ± 0.008	8 (8–12)	3.04
*Heliotropium megalanthum–Heliotropium longistylum*	0.2362	0.218 ± 0.015	4 (4–4)	1.52
*Heliotropium megalanthum–Heliotropium sinuatum*	0.2498	0.221 ± 0.010	9 (9–11)	3.42
*Heliotropium longistylum–Heliotropium sinuatum*	0.2288	0.196 ± 0.012	7 (5–7)	2.66

## Discussion

Our results reveal a number of previously unknown findings regarding the five sympatric species of *Heliotropium* sect. *Cochranea* included in this study. First, the obtained phylogenetic relationships are well supported and consistent with previous works (Luebert and Wen 2008; Luebert et al. 2011a,b2011b). However, the present analysis resolves a well-supported sister relationship between *H. krauseanum* and all other species except *H. pycnophyllum* (which is sister to all other species of this group). This relationship was not resolved in previous analyses (Luebert and Wen 2008; Luebert et al. 2011a). Second, the five sympatric species seem to be genetically well differentiated from each other in spite of their relatively recent origin (Luebert and Wen 2008; Luebert et al. 2011b): They occupy distinct regions in the genetic space and, with the exception of *H. floridum* versus *H. megalanthum* (*F*_ST_* *= 0.1004), all species-pair comparisons have *F*_ST_ estimates larger than 0.17 (see Table 4). This is reinforced by the fact that a considerable proportion of the total genetic differentiation (∼35%) is due to among-species variation. Figure 1B shows that genetic distance tends to be highest between *H. filifolium* and the other *Heliotropium* species, which is consistent with its phylogenetic position outside of a poorly resolved clade in which the remaining species are located (Fig. 1D). However, sister relationships within this clade remain unknown.

A few individuals that were morphologically assigned to a certain species clustered together with individuals of another species. Barring identification errors, this may indicate that, despite genetic differentiation among species, gene flow still occurs between the involved species. In fact, measures of gene flow calculated according to the finite island model also suggest that these species experience different levels of gene flow and that levels of gene flow are correlated with phylogenetic relatedness. This picture is consistent with personal field observations on these sympatric species that indicate clear morphological differentiation (Luebert 2013). Differentiation might be associated with different strategies to tolerate drought (Luebert et al. 2011a) and how species are pollinated (Luebert 2013). In the latter sense, relative position and size of reproductive floral structures may be associated with different pollinators, a hypothesis that remains to be tested. Additionally, hybrids may occur in the areas of sympatry. In support of this, morphologically intermediate individuals between *H. sinuatum* and *H. longistylum,* between *H. longistylum* and *H. floridum,* and between *H. floridum* and *H. maegalanthum* have been observed (Luebert 2013; pers. obs.). Field annotations are also consistent with the observed patterns of genetic differentiation. For example, the sample Fili7 was annotated in the field to be a putative hybrid between *H. filifolium* and *H. sinuatum*, because it exhibited floral morphological characters of the former species, but leaf characters transitional to the latter. Our AFLP data suggest that this individual falls within the genetic space of *H. sinuatum* (see Fig. 1A).

Comparisons of pairs of sympatric species with DOA indicate that few of the investigated loci are expected to be under positive selection. This is a signal that allopatric speciation may not have taken place among these closely related species: Under such a scenario, it is expected that a large portion of the genome diverges by a combination of selection and genetic drift and that the number of divergent portions of the genomes increase with time since speciation (Wu 2001; Nosil et al. 2009), which is not the case here. Allopatric speciation should also be characterized by homogeneous divergence across the genome (Wu and Ting 2004). Our data tend to support a model of nonallopatric speciation in which divergence takes place in the presence of gene flow (Nosil et al. 2009; Strasburg et al. 2012). As absence of gene flow appears to be restricted to only a few loci, speciation in *Heliotropium* sect. *Cochranea* is probably an incipient and ongoing process (Wu 2001). Whether speciation with gene flow did occur in *Heliotropium* sect. *Cochranea* remains to be seen when more detailed phylogenetic and population data become available, but the data presented in this study are at least in line with such a scenario. However, if the duration of the periods of allopatry were relatively short and populations came relatively early during the speciation process into secondary contact, the signature of the DOA may be indistinguishable from divergence with gene flow (Strasburg et al. 2012), in part because gene flow will tend to homogenize polymorphisms that may have accumulated during periods of allopatry in those loci that are not subject to selection (Via and West 2008). Luebert and Wen (2008) postulated a model of diversification of *Heliotropium* sect. *Cochranea* in which divergence alternates with recombination in periods of contraction and expansion of population ranges (Stebbins 1952) associated with fluctuations of aridity in the Atacama Desert. Drier periods would have promoted contraction of population ranges and therefore made allopatry more likely. Conversely, more humid periods would have favoured population expansion, thus increasing the probability of secondary contact. If pulses of arid and humid periods in the Atacama Desert are short and produce the effect described by Stebbins (1952), then the predictions of Luebert and Wen (2008) will be compatible with the data presented in this study.

Climatic and paleoecological studies in the Atacama Desert suggest that arid and humid periods have alternated at different timescales. This occurs as a consequence of the El Niño phenomenon and the Interdecadal Pacific Oscillation (annual to decadal timescales; Dillon and Rundel 1990; Schulz et al. 2012), as well as geographically larger scale fluctuations associated with sea surface temperatures (millennial timescales; Latorre et al. 2006; Placzek et al. 2009; Gayo et al. 2012) and even larger timescales related to the glacial periods (Haselton et al. 2002) or other geological or climatic events such as the Andean uplift or the effect of the Humboldt Current (Hartley 2003). While most evolutionary studies on Atacama Desert plants have focused on large-scale fluctuations and allopatry was therefore the preferred mechanism to explain diversification (Gengler-Nowak 2002; Luebert and Wen 2008; Luebert et al. 2009; Viruel et al. 2012), this study suggests that short-time fluctuations and periods of sympatry may also play a role in the evolutionary diversification of plants in the Atacama Desert.

Premating isolation mechanisms might be at play if floral morphology is a good indicator of pollination preferences in *Heliotropium*. Floral morphology in *Heliotropium* (Hilger 1987, 1989) is characterized by a conical stigmatic head with a basal receptive tissue. A nectar disk is located at base of the ovary. The gynoecium is surrounded by five stamens in the tube of a infundibuliform corolla, leaving limited space to a pollinator to reach the nectar. This floral architecture suggests insect pollination, and insect pollination has been shown to occur in *Heliotropium* (e.g., Grant 1949; Weiss 1991; McMullen 2007). In *Hancornia speciosa* Gomes (Apocynaceae), a species with similar flower morphology to *Heliotropium* Darrault and Schlindwein (2005) showed that only insects with matching proboscis lengths are able to pollinate. Corolla tube length, relative position of the statements in the tube, and ratio between length of the style and length of the stigma vary across sympatric species of *Heliotropium* sect. *Cochranea*, but are relatively stable within populations. This can be an indication that different species are adapted to different pollinators (Luebert 2013). However, whether floral morphology has an actual effect on pollination in *Heliotropium* is an issue that needs to be assessed in the future.

## Data archiving

DNA sequences deposited in GenBank: Accessions KF301622–KF301628. TreeBase: accession 14422. Dryad, DOI: 10.5061/dryad.ps46g

## Supporting Information

Figure S1 Graphic representation of the Divergence Outlier Analysis for each species pair obtained from Mcheza.

Table S1 Individuals of which DNA was employed in the AFLP analysis of *Heliotropium* sect. *Cochranea*.

Table S2 Voucher specimen information and GenBank accession numbers of the sequences of *Heliotropium* sect. *Cochranea*.

Table S3 Phylogenetic distance (PD), genetic differentiation (F_ST_), and gene flow estimated as number of migrants per generation (N_e_m) between each pair of species of Heliotropium sect. *Cochranea*.
