# Supplementary material for: Evidence for nonallopatric speciation among closely related sympatric Heliotropium species in the Atacama Desert
Source: Ecol Evol. 2013 Dec 29;4(3):266–75. doi: 10.1002/ece3.929 (PMC3925428; doi:10.1002/ece3.929)
Supplement: Figure S1 — Graphic representation of the Divergence Outlier Analysis for each species pair obtained from Mcheza. [file ece30004-0266-sd1.pdf]

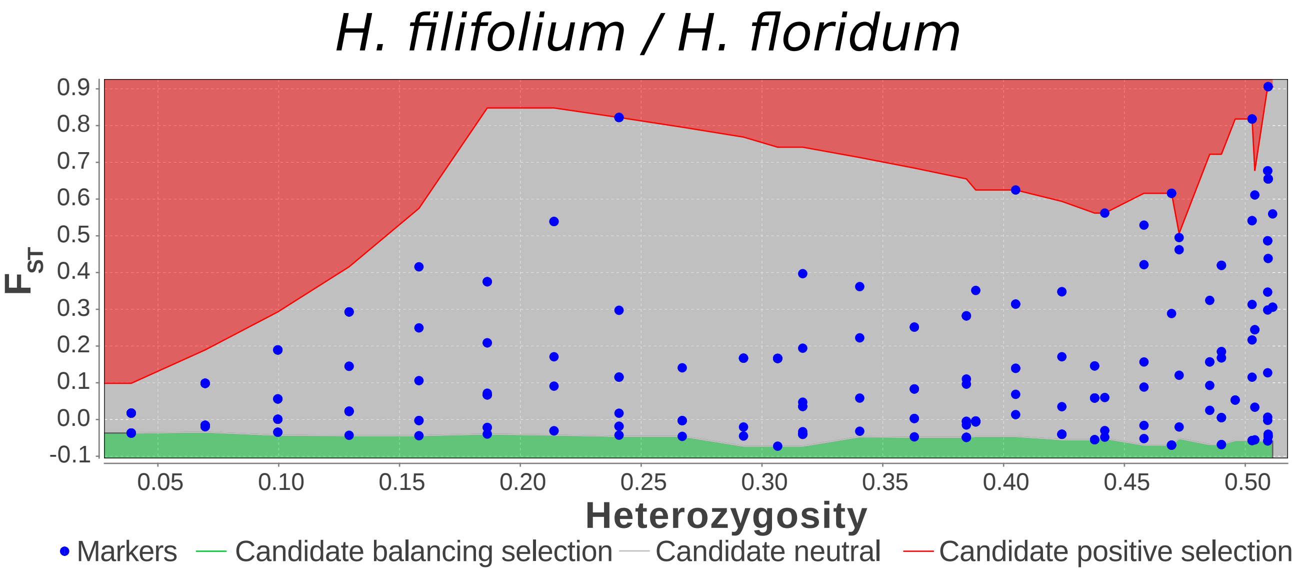
H. filifolium / H. floridum
F_{ST}
0.9
0.8
0.7
0.6
0.5
0.4
0.3
0.2
0.1
0.0
-0.1
0.05
0.10
0.15
0.20
0.25
0.30
0.35
0.40
0.45
0.50
Heterozygosity
Markers
Candidate balancing selection
Candidate neutral
Candidate positive selection

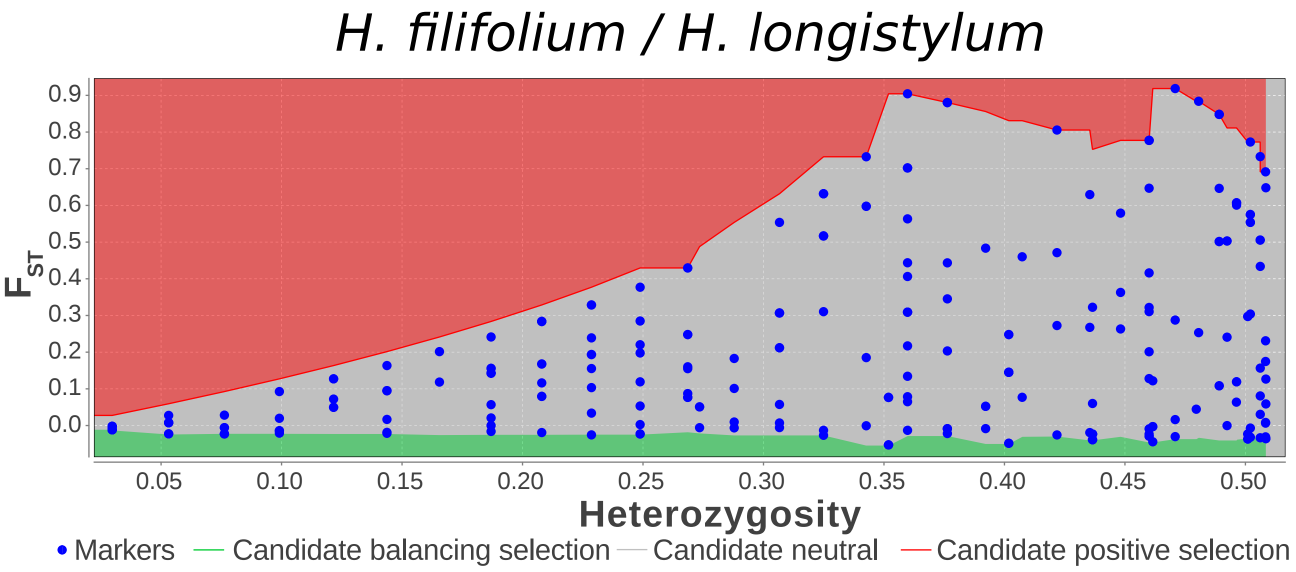
H. filifolium / H. longistylum
F_{ST}
0.9
0.8
0.7
0.6
0.5
0.4
0.3
0.2
0.1
0.0
0.05
0.10
0.15
0.20
0.25
0.30
0.35
0.40
0.45
0.50
Heterozygosity
Markers
Candidate balancing selection
Candidate neutral
Candidate positive selection

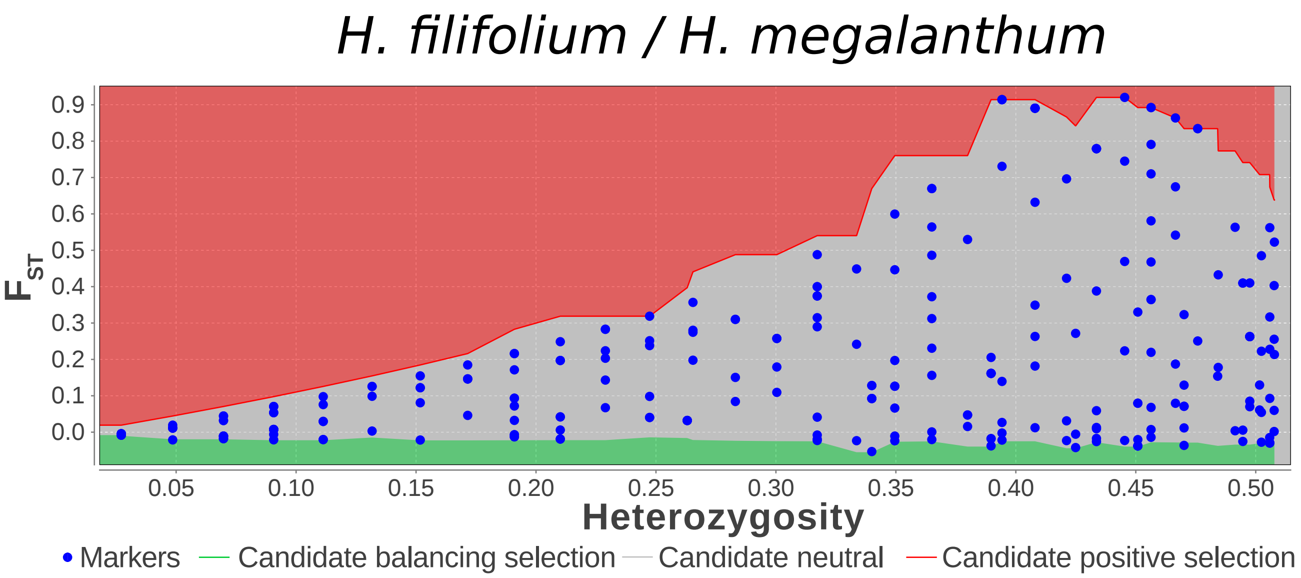
H. filifolium / H. megalanthum
F_{ST}
0.9
0.8
0.7
0.6
0.5
0.4
0.3
0.2
0.1
0.0
0.05
0.10
0.15
0.20
0.25
0.30
0.35
0.40
0.45
0.50
Heterozygosity
Markers
Candidate balancing selection
Candidate neutral
Candidate positive selection

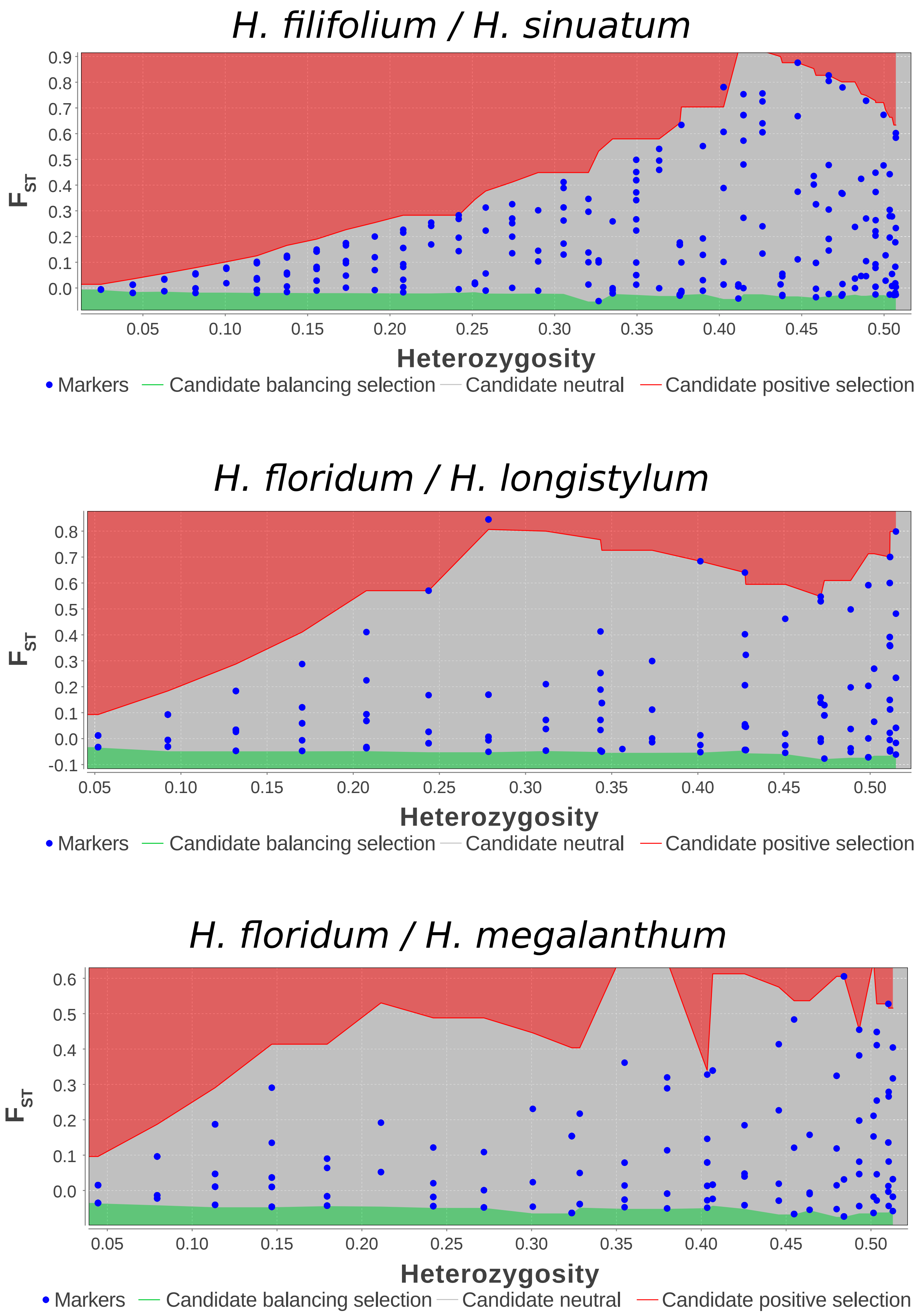

H. filifolium / H. sinuatum
F_ST
Heterozygosity
Markers
Candidate balancing selection
Candidate neutral
Candidate positive selection
H. floridum / H. longistylum
F_ST
Heterozygosity
Markers
Candidate balancing selection
Candidate neutral
Candidate positive selection
H. floridum / H. megalanthum
F_ST
Heterozygosity
Markers
Candidate balancing selection
Candidate neutral
Candidate positive selection

H. floridum / H. sinuatum

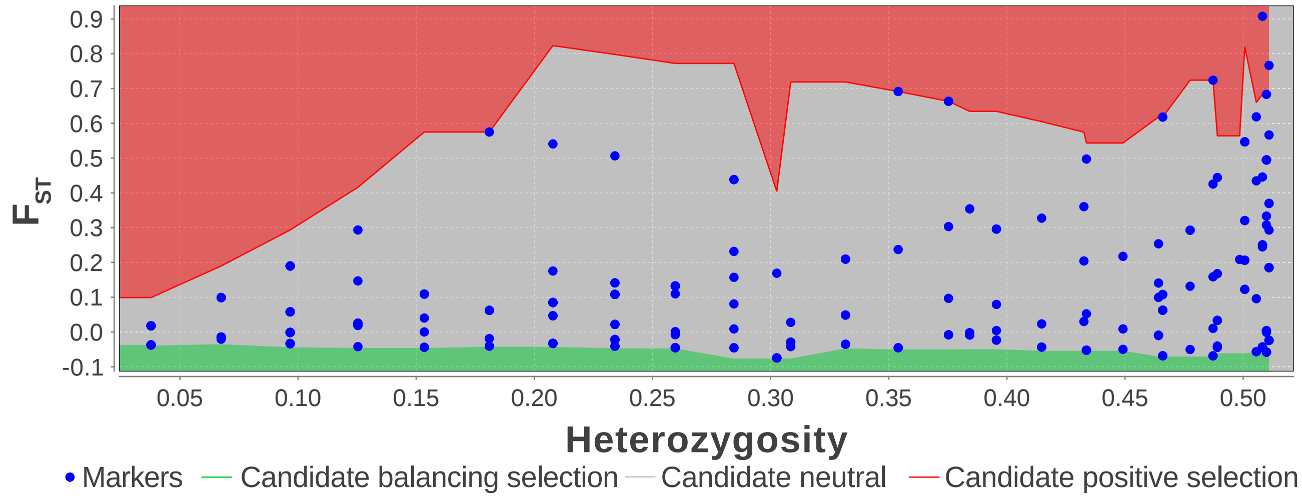

H. longistylum / H. megalanthum

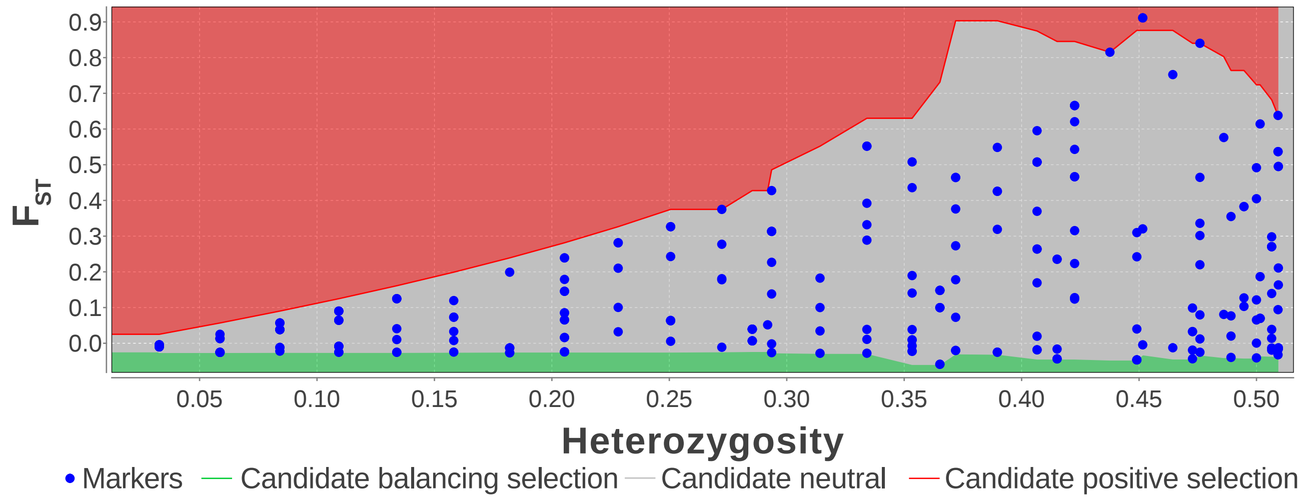

H. longistylum / H. sinuatum

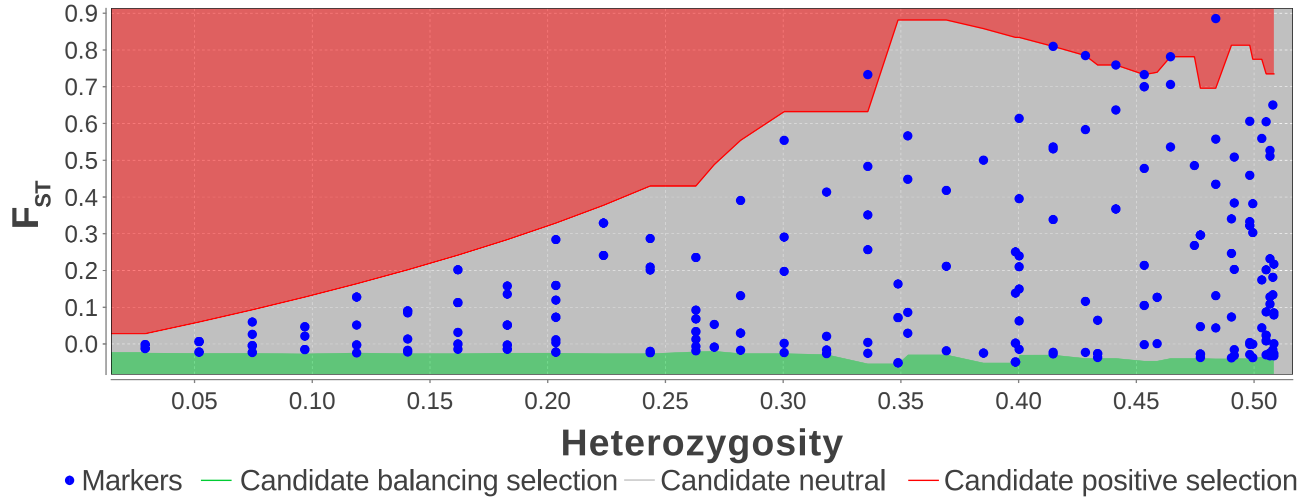

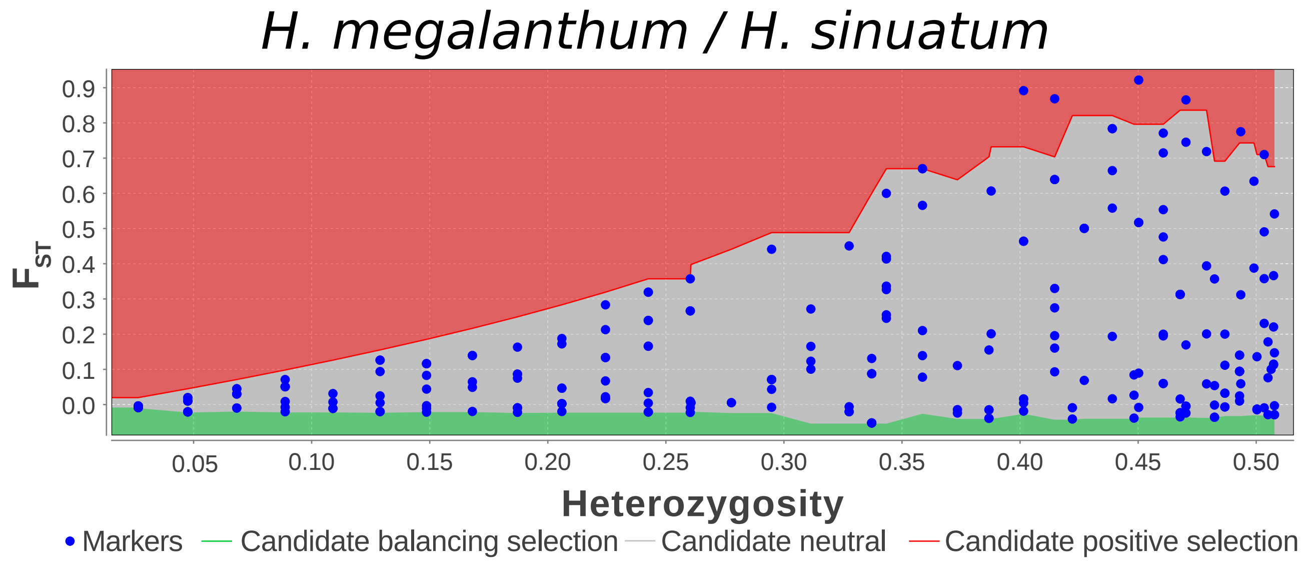
H. megalanthum / H. sinuatum
F_ST
0.9
0.8
0.7
0.6
0.5
0.4
0.3
0.2
0.1
0.0
0.05
0.10
0.15
0.20
0.25
0.30
0.35
0.40
0.45
0.50
Heterozygosity
Markers
Candidate balancing selection
Candidate neutral
Candidate positive selection
