# Supplementary material for: Evidence for nonallopatric speciation among closely related sympatric Heliotropium species in the Atacama Desert
Source: Ecol Evol. 2013 Dec 29;4(3):266–75. doi: 10.1002/ece3.929 (PMC3925428; doi:10.1002/ece3.929)
Supplement: Table S1 — Individuals of which DNA was employed in the AFLP analysis of Heliotropium sect. Cochranea. [file ece30004-0266-sd2.doc]

Table S1. Individuals of which DNA was employed in the AFLP analysis of *Heliotropium* sect. *Cochranea*, indicating species, collection year (Year), Number of individuals (Nind), collection locality, voucher specimen (collector, collection number, herbarium code) and samples codes referred to in this study. Herbarium codes: SGO, National Museum of Natural History, Santiago, Chile; BSB: Institute of Biology – Botany, Freie University Berlin, Germany.

| Species | Year | Nind | Locality | Voucher specimen | Sample codes |
| --- | --- | --- | --- | --- | --- |
| *Heliotropium longistylum* | 2011 | 15 | Totoral | *Luebert 3058* (SGO) | Long01 – Long30 |
| *Heliotropium longistylum* | 2004 | 1 | Totoral | *Luebert et al. 2020* (SGO) | Long34 |
| *Heliotropium longistylum* | 2003 | 1 | Carrizal Bajo | *Luebert & Kritzner 1811* (BSB) | Long36 |
| *Heliotropium filifolium* | 2011 | 23 | Totoral | *Luebert 3048* (SGO) | Fili01 – Fili32 |
| *Heliotropium filifolium* | 2004 | 2 | Totoral | *Luebert et al. 2015* (SGO) | Fili34, Fili36 |
| *Heliotropium megalanthum* | 2011 | 18 | Totoral | *Luebert 3057* (SGO) | Mega01 – Mega22 |
| *Heliotropium megalanthum* | 2004 | 3 | Totoral | *Luebert et al. 2019* (BSB) | Mega23 – Mega26 |
| *Heliotropium sinuatum* | 2011 | 25 | Totoral | *Luebert 3047* (SGO) | Sinu01 – Sinu36 |
| *Heliotropium sinuatum* | 2005 | 1 | Carrizal Bajo | *Luebert & Becker 2899* (BSB) | Sinu40 |
| *Heliotropium floridum* | 2004 | 4 | Totoral | *Luebert et al. 2031* (SGO) | Flori01 – Flori04 |
| *Heliotropium floridum* | 2003 | 1 | Carrizal Bajo | *Luebert & Krtizner 1801* (BSB) | Flori06 |
| *Heliotropium floridum* | 2003 | 1 | Carrizal Bajo | *Luebert & Krtizner 1810* (BSB) | Flori07 |
| *Heliotropium floridum* | 2003 | 1 | Carrizal Bajo | *Luebert & Krtizner 1819* (BSB) | Flori08 |
