# Supplementary material for: Evidence for nonallopatric speciation among closely related sympatric Heliotropium species in the Atacama Desert
Source: Ecol Evol. 2013 Dec 29;4(3):266–75. doi: 10.1002/ece3.929 (PMC3925428; doi:10.1002/ece3.929)
Supplement: Table S2 — Voucher specimen information and GenBank accession numbers of the sequences of Heliotropium sect. Cochranea. [file ece30004-0266-sd3.doc]

Table S2. Voucher specimen information and GenBank accession numbers of the sequences of *Heliotropium* sect. *Cochranea* used in the phylogenetic analysis.

| Species | Voucher specimen | Reference | Isolate | Group | ITS | ndhF | rps16 | trnL-F |
| --- | --- | --- | --- | --- | --- | --- | --- | --- |
| *Heliotropium chenopodiaceum* | Luebert & García 2501 (SGO) | Luebert & Wen (2008) | FL35 | Cochranea | EF688872 | EF688924 | EF688975 | EF688819 |
| *Heliotropium chenopodiaceum* | Luebert & García 2462 (SGO) | Luebert & Wen (2008) | FL32 | Cochranea | EF688869 | EF688921 | EF688972 | EF688816 |
| *Heliotropium chenopodiaceum* | Luebert & Becker 2907 (SGO) | Luebert & Wen (2008) | FL25 | Cochranea | EF688864 | EF688916 | EF688967 | EF688811 |
| *Heliotropium eremogenum* | Luebert & García 2575 (SGO) | Luebert & Wen (2008) | FL28 | Cochranea | EF688865 | EF688917 | EF688968 | EF688812 |
| *Heliotropium eremogenum* | Luebert et al. 2159 (SGO) | Luebert & Wen (2008) | FL44 | Cochranea | EF688880 | EF688933 | EF688983 | EF688827 |
| *Heliotropium filifolium* | Luebert & Torres 1973 (SGO) | Luebert & Wen (2008) | FL46 | Cochranea | EF688882 | EF688935 | EF688985 | EF688829 |
| *Heliotropium filifolium* | Luebert et al. 2015 (SGO) | Luebert & Wen (2008) | FL70 | Cochranea | EF688900 | EF688951 | EF689002 | EF688848 |
| *Heliotropium floridum* | Luebert & Torres 1974 (SGO) | Luebert & Wen (2008) | FL48 | Cochranea | EF688884 | EF688937 | EF688987 | EF688831 |
| *Heliotropium floridum* | Luebert & Becker 2838 (SGO) | Luebert & Wen (2008) | FL21 | Cochranea | EF688861 | EF688913 | EF688964 | EF688808 |
| *Heliotropium floridum* | Luebert et al. 2031 (SGO) | Luebert & Wen (2008) | FL58 | Cochranea | EF688893 | EF688946 | EF688996 | EF688840 |
| *Heliotropium glutinosum* | Luebert Torres 1970 (SGO) | Luebert & Wen (2008) | FL50 | Cochranea | EF688885 | EF688938 | EF688988 | EF688832 |
| *Heliotropium glutinosum* | Luebert & Beckler 2161 (SGO) | Luebert & Wen (2008) | FL43 | Cochranea | EF688879 | EF688932 | EF688982 | EF688826 |
| *Heliotropium inconspicuum* | Luebert et al. 2081 (SGO) | Luebert & Wen (2008) | FL56 | Cochranea | EF688891 | EF688944 | EF688994 | EF688838 |
| *Heliotropium inconspicuum* | Luebert et al. 2095 (SGO) | Luebert & Wen (2008) | FL55 | Cochranea | EF688890 | EF688943 | EF688993 | EF688837 |
| *Heliotropium inconspicuum* | Luebert & García 2783 (SGO) | Luebert & Wen (2008) | FL22 | Cochranea | EF688862 | EF688914 | EF688965 | EF688809 |
| *Heliotropium krauseanum* | Dillon 8779 (F) | Luebert & Wen (2008) | FL59 | Cochranea | EF688841 | EF688947 | EF688997 | EF688894 |
| *Heliotropium krauseanum* | Weigend 97/727 (B) |  | 233 | Cochranea | KF301622 | KF301623 | KF301626 | KF301628 |
| *Heliotropium linariaefolium* | Luebert & García 2731 (SGO) | Luebert & Wen (2008) | FL23 | Cochranea | EF688863 | EF688915 | EF688966 | EF688810 |
| *Heliotropium linariaefolium* | Luebert & Becker 2844A (SGO) | Luebert & Wen (2008) | FL33 | Cochranea | EF688870 | EF688922 | EF688973 | EF688817 |
| *Heliotropium linariaefolium* | Luebert et al. 2054 (SGO) | Luebert & Wen (2008) | FL57 | Cochranea | EF688892 | EF688945 | EF688995 | EF688839 |
| *Heliotropium longistylum* | Luebert & Torres 1971 (SGO) | Luebert & Wen (2008) | FL47 | Cochranea | EF688883 | EF688936 | EF688986 | EF688830 |
| *Heliotropium longistylum* | Luebert et al. 2020 (SGO) | Luebert & Wen (2008) | FL71 | Cochranea | EF688901 | EF688952 | EF689003 | EF688849 |
| *Heliotropium megalanthum* | Luebert & Becker 2165 (SGO) | Luebert & Wen (2008) | FL40 | Cochranea | EF688876 | EF688929 | EF688979 | EF688823 |
| *Heliotropium megalanthum* | Teillier s.n. (SGO) | Luebert & Wen (2008) | FL63 | Cochranea | EF688897 | KF301624 | KF301627 | EF688844 |
| *Heliotropium myosotifolium* | Luebert & Becker 2162 (SGO) | Luebert & Wen (2008) | FL42 | Cochranea | EF688878 | EF688931 | EF688981 | EF688825 |
| *Heliotropium myosotifolium* | Luebert et al. 2011 (SGO) | Luebert et al. (2011) | FL72 | Cochranea | HQ286107 | KF301625 | HQ286228 | HQ286135 |
| *Heliotropium philippianum* | Luebert et al. 2124 (SGO) | Luebert & Wen (2008) | FL52 | Cochranea | EF688887 | EF688940 | EF688990 | EF688834 |
| *Heliotropium philippianum* | Luebert et al. 2131 (SGO) | Luebert & Wen (2008) | FL51 | Cochranea | EF688886 | EF688939 | EF688989 | EF688833 |
| *Heliotropium pycnophyllum* | Luebert & García 2620 (SGO) | Luebert & Wen (2008) | FL29 | Cochranea | EF688866 | EF688918 | EF688969 | EF688813 |
| *Heliotropium pycnophyllum* | Luebert & García 2813 (SGO) | Luebert & Wen (2008) | FL31 | Cochranea | EF688868 | EF688920 | EF688971 | EF688815 |
| *Heliotropium sinuatum* | Luebert & Torres 1972 (SGO) | Luebert & Wen (2008) | FL45 | Cochranea | EF688881 | EF688934 | EF688984 | EF688828 |
| *Heliotropium sinuatum* | Luebert & García 2492 (SGO) | Luebert & Wen (2008) | FL34 | Cochranea | EF688871 | EF688923 | EF688974 | EF688818 |
| *Heliotropium stenophyllum* | Luebert & Becker 2902 (SGO) | Luebert & Wen (2008) | FL36 | Cochranea | EF688873 | EF688925 | EF688976 | EF688820 |
| *Heliotropium stenophyllum* | Luebert & Becker 2168 (SGO) | Luebert & Wen (2008) | FL41 | Cochranea | EF688877 | EF688930 | EF688980 | EF688824 |
| *Heliotropium stenophyllum* | Luebert & Becker 2909 (SGO) | Luebert & Wen (2008) | FL37 | Cochranea | EF688874 | EF688926 | EF688977 | EF688821 |
| *Heliotropium stenophyllum* | Luebert & Becker 1990 (SGO) | Luebert & Wen (2008) | FL69 | Cochranea | EF688899 | EF688950 | EF689001 | EF688847 |
| *Heliotropium stenophyllum* | Luebert & Becker 2911 (SGO) | Luebert & Wen (2008) | FL39 | Cochranea | EF688875 | EF688928 | EF688978 | EF688822 |
| *Heliotropium taltalense* | Luebert & García 2650 (SGO) | Luebert & Wen (2008) | FL30 | Cochranea | EF688867 | EF688919 | EF688970 | EF688814 |
| *Heliotropium taltalense* | Luebert et al. 2101 (SGO) | Luebert & Wen (2008) | FL53 | Cochranea | EF688888 | EF688941 | EF688991 | EF688835 |
| *Heliotropium taltalense* | Luebert et al. 2083 (SGO) | Luebert & Wen (2008) | FL54 | Cochranea | EF688889 | EF688942 | EF688992 | EF688836 |
| *Heliotropium nicotianifolium* | Nee & Wen 53890 (US) | Luebert & Wen (2008) | FL6 | Outgroup | EF688857 | EF688909 | EF688960 | EF688804 |
| *Heliotropium nicotianifolium* | Nee & Wen 53843 (US) | Luebert & Wen (2008) | FL3 | Outgroup | EF688854 | EF688906 | EF688957 | EF688801 |
| *Heliotropium paronychioides* | Luebert & Teillier 2241 (SGO) | Luebert & Wen (2008) | FL60 | Outgroup | EF688895 | EF688948 | EF688998 | EF688842 |
| *Heliotropium elongatum* | Nee & Wen 53844 (US) | Luebert & Wen (2008) | FL4 | Outgroup | EF688855 | EF688907 | EF688958 | EF688802 |
| *Tournefortia* cf. *buchtienii* | Nee & Wen 53944 (US) | Luebert & Wen (2008) | FL7 | Outgroup | EF688858 | EF688910 | EF688961 | EF688805 |
| *Heliotropium curassavicum* var. *curassavicum* | Luebert & García 2521 (SGO) | Luebert & Wen (2008) | FL61 | Outgroup | EF688896 | EF688949 | EF688999 | EF688843 |
| *Heliotropium arborescens* var. *grisellum* | Dillon 8838 (F) | Luebert & Wen (2008) | FL19 | Outgroup | EF688859 | EF688911 | EF688962 | EF688806 |
| *Myriopus rubicundus* | Nee & Wen 53846 (US) | Luebert & Wen (2008) | FL1 | Outgroup | EF688852 | EF688904 | EF688955 | EF688799 |
| *Myriopus salzmannii* | Nee & Wen 53848 (US) | Luebert & Wen (2008) | FL2 | Outgroup | EF688853 | EF688905 | EF688956 | EF688800 |
| *Euploca procumbens* | Nee & Wen 53873 (US) | Luebert & Wen (2008) | FL5 | Outgroup | EF688856 | EF688908 | EF688959 | EF688803 |
| *Euploca pilosa* | Dillon 8819 (F) | Luebert & Wen (2008) | FL73 | Outgroup | EF688902 | EF688953 | EF689004 | EF688850 |
| *Tiquilia paronychioides* | Dillon 8798 (F) | Luebert & Wen (2008) | FL20 | Outgroup | EF688860 | EF688912 | EF688963 | EF688807 |
| *Cordia decandra* | Luebert & Kritzner 1873 (SGO) | Luebert & Wen (2008) | FL74 | Outgroup | EF688903 | EF688954 | EF689005 | EF688851 |
