# Supplementary material for: Evidence for nonallopatric speciation among closely related sympatric Heliotropium species in the Atacama Desert
Source: Ecol Evol. 2013 Dec 29;4(3):266–75. doi: 10.1002/ece3.929 (PMC3925428; doi:10.1002/ece3.929)
Supplement: Table S3 — Phylogenetic distance (PD), genetic differentiation (FST), and gene flow estimated as number of migrants per generation (Nem) between each pair of species of Heliotropium sect. Cochranea. [file ece30004-0266-sd4.doc]

Table S3. Phylogenetic distance (PD), genetic differentiation (*F_ST_*) and gene flow estimated as number of migrants per generation (N_e_m) between each pair of species of *Heliotropium* sect. *Cochranea* in the area of Totoral, Chile.

| Species pair | PD | *F_ST_* | N_e_m |
| --- | --- | --- | --- |
| *H. filifolium - H. floridum* | 0.0189 | 0.3445 | 0.4757 |
| *H. filifolium - H. megalanthum* | 0.0189 | 0.4185 | 0.3474 |
| *H. filifolium - H. longistylum* | 0.0189 | 0.4236 | 0.3402 |
| *H. filifolium - H. sinuatum* | 0.0189 | 0.3872 | 0.3957 |
| *H. floridum - H. megalanthum* | 0.0030 | 0.1391 | 1.5475 |
| *H. floridum - H. longistylum* | 0.0081 | 0.2424 | 0.7815 |
| *H. floridum - H. sinuatum* | 0.0081 | 0.2489 | 0.7543 |
| *H. megalanthum - H. longistylum* | 0.0081 | 0.3320 | 0.5030 |
| *H. megalanthum - H. sinuatum* | 0.0081 | 0.3375 | 0.4907 |
| *H. longistylum - H. sinuatum* | 0.0035 | 0.3130 | 0.5487 |
